# High-mobility group box 1 potentiates antineutrophil cytoplasmic antibody-inducing neutrophil extracellular traps formation

**DOI:** 10.1186/s13075-015-0903-z

**Published:** 2016-01-06

**Authors:** Yun-Hua Ma, Tian-tian Ma, Chen Wang, Huan Wang, Dong-Yuan Chang, Min Chen, Ming-Hui Zhao

**Affiliations:** Renal Division, Department of Medicine, Peking University First Hospital, Peking University Institute of Nephrology, Beijing, 100034 China; Key Laboratory of Renal Disease, Ministry of Health of China, Beijing, 100034 China; Key Laboratory of Chronic Kidney Disease Prevention and Treatment, Peking University, Ministry of Education, Beijing, 100034 China; Peking-Tsinghua Center for Life Sciences, Beijing, 100034 China; Renal Division, Department of Medicine, First Affiliated Hospital of Guangxi Medical University, Nanning, Guangxi Zhuang Autonomous Region 530021 China

**Keywords:** High-mobility group box 1, ANCA, Neutrophil extracellular traps

## Abstract

**Background:**

Recent studies found that the circulating high-mobility group box 1 (HMGB1) levels could reflect the disease activity of antineutrophil cytoplasmic antibody (ANCA)-associated vasculitis (AAV). HMGB1 could prime neutrophils by increasing ANCA antigens translocation for ANCA-mediated respiratory burst and degranulation. The current study aimed to investigate whether HMGB1 participates in ANCA-induced neutrophil extracellular traps (NETs) formation, which is one of the most important pathogenic aspects in the development of AAV.

**Methods:**

NETs were induced by treating neutrophils with HMGB1 and ANCA-positive IgG in vitro. NETs formation was assessed using immunofluorescence microscopy and fluorescence probe. Antagonist for relevant receptors Toll-like receptor (TLR)2, TLR4 and the receptor for advanced glycation end products (RAGE), as well as NADPH oxidase molecules were employed.

**Results:**

The percentage of NETs formation was significantly higher in neutrophils stimulated with HMGB1 plus ANCA-positive IgG than that in neutrophils incubated with HMGB1 or ANCA-positive IgG alone. Consistently, compared with the nonstimulated neutrophils, the cell-free DNA (cfDNA) concentration of NETs was significantly increased from 334.09 ± 46.89 ng/ml to 563.32 ± 122.07 ng/ml in the neutrophils incubated with HMGB1 plus MPO-ANCA-positive IgG (*P* < 0.001), and from 303.44 ± 37.14 ng/ml to 563.79 ± 145.94 ng/ml in the neutrophils incubated with HMGB1 plus PR3-ANCA-positive IgG (*P* < 0.001). The aforementioned effect was significantly attenuated by antagonist for relevant receptors TLR2, TLR4 and RAGE, as well as blocking NADPH oxidase.

**Conclusions:**

HMGB1 can potentiate ANCA-inducing NETs formation and may be involved in the pathogenesis of AAV. HMGB1 exerts effects on NETs formation through interaction with TLR2, TLR4 and RAGE, and the process is NADPH oxidase dependent.

**Electronic supplementary material:**

The online version of this article (doi:10.1186/s13075-015-0903-z) contains supplementary material, which is available to authorized users.

## Background

Antineutrophil cytoplasmic antibody (ANCA)-associated vasculitis (AAV) encompasses granulomatosis with polyangiitis (GPA) and eosinophilic granulomatosis with polyangiitis (EGPA) and microscopic polyangiitis (MPA) [1]. The serological markers for the aforementioned primary small vessel vasculitis were ANCAs, which direct against granule proteins of neutrophils, in particular, proteinase 3 (PR3) and myeloperoxidase (MPO). Accumulated evidence from in vitro studies, animal models, and clinical observations suggests that ANCAs play a critical role in the vascular damage of AAV [2, 3].

Neutrophils are the primary effector cells in the pathogenesis of AAV [4]. Cytokines or other pro-inflammatory mediators, such as tumor necrosis factor alpha (TNF-α) and interleukin (IL)-18, could prime neutrophils by inducing upregulation of the ANCA antigens expression on the surface of neutrophils. Thus, ANCAs could further activate primed neutrophils to undergo a respiratory burst and degranulation, which plays a crucial role in the development of vasculitis [5, 6]. Recently, neutrophil extracellular traps (NETs), generated by ANCA-activated neutrophils, are increasingly recognized as another important aspect in the pathogenesis of AAV [7, 8]. NETs consist of decondensed chromatin modification with cytoplasmic proteins such as MPO and PR3, which are induced by a wide range of stimuli including pathogens (bacteria, fungi), activated platelets and cytokines [8, 9]. NETs can stick to the endothelium and cause tissue damage during inflammation similar to neutrophil-induced injury of capillaries in AAV [10]. Recent studies showed that the abnormal regulation of NETs is involved in the pathogenesis of AAV [7, 11]. On the other hand, NETs are associated with thrombosis in AAV patients because histones and DNA within NETs can bind platelets and blood coagulants [12, 13].

High-mobility group box 1 protein (HMGB1), a ubiquitous nuclear protein involved in nucleosome stabilization and gene transcription, has potent pro-inflammatory actions and can be classified as a danger-associated molecular pattern mediator when placed extracellularly [14]. HMGB1 can interact with Toll-like receptor (TLR)-2, TLR-4 and the receptor for advanced glycation end products (RAGE) in established cell lines and animal models, leading to a downstream translocation of nuclear factor (NF)-κB, inducing pro-inflammatory and chemotactic responses [15, 16].

Recent studies found that circulating HMGB1 levels are associated the disease activity and renal involvement of AAV [17–19], although it remains controversial in some other studies [20]. Our further study found that HMGB1 could prime neutrophils by increasing ANCA antigens translocation, and the primed neutrophils could be further induced by ANCA, resulting in the respiratory burst and degranulation [21]. Therefore, in the current study, we hypothesized that HMGB1 can contribute to ANCA-induced NETs formation. Furthermore, we also investigated receptors and signaling molecules involved in HMGB1-promoted NETs formation in the presence of ANCA.

## Methods

### Reagent

For immunofluorescence analysis, rabbit polyclonal anti-Cit-histone H3 antibody and mouse monoclonal anti-human myeloperoxidase antibody were purchased from Abcam (Cambridge, UK), Alexa Fluor 488 donkey anti-rabbit IgG and Cy3-labeled donkey anti-mouse IgG were purchased from Jackson ImmunoResearch (West Grove, PA, USA), and 4′,6-diamidino-2-phenylindole (DAPI) was purchased from Zhongshan Golden Bridge Biotechnology (Beijing, China). Phorbol myristate acetate (PMA) and diphenyleneiodonium (DPI) were purchased from Sigma-Aldrich (St. Louis, MO, USA). Purified anti-human CD282 (TLR2) and CD284 (TLR4) were purchased from BioLegend (San Diego, CA, USA). Recombinant HMGB1 proteins were purchased from R&D Systems (C23 and C45 disulfide C106 thiol form) (Abingdon, UK). RAGE-Fc was purchased from R&D Systems (Minneapolis, MN, USA).

### Preparation of IgG

Normal immunoglobulin G (IgG) and ANCA-positive IgG were prepared from plasma of normal volunteers and patients with active PR3-ANCA- or MPO-ANCA-positive primary small vessel vasculitis. Plasma was filtered through a 0.22 mm syringe filter (Gelman Sciences, Ann Arbor, MI, USA) and applied to a HiTrap Protein G column on an AKTA-FPLC system (GE Biosciences, South San Francisco, CA, USA). Preparation of IgG was performed according to the methods described previously [22, 23].

### Neutrophil isolation

Neutrophils were isolated as described previously [24]. Briefly speaking, venous human blood for neutrophil isolation was obtained from healthy donors by venipuncture and anticoagulated with EDTA. Neutrophils were isolated by density gradient centrifugation on Lymphoprep (Nycomed, Oslo, Norway). Erythrocytes were lysed with ice-cold ammonium chloride buffer, and neutrophils were washed with phosphate-buffered saline (PBS) (Beijing Chemical Reagents, Beijing, China). The purity of the neutrophils was above 95 %. Neutrophils were then suspended in RPMI 1640 containing 0.5 % heat-inactivated fetal bovine serum (FBS). We obtained written informed consent from all participants. The research was in compliance of the Declaration of Helsinki and approved by the ethics committee of the Peking University First Hospital.

### NETs induction

NETs induction was according to the protocols described previously, with some minor modifications [25, 26]. Isolated neutrophils were seeded on 13 mm glass coverslips in 24-well plates in 400 μl of RPMI 1640 supplemented with 0.5 % heat-inactivated FBS at a density of 1 × 10^5^ cells per well. The plates were incubated for 30–60 min at 37 °C to allow adhesion of the cells. Then neutrophils were incubated with the buffer control or HMGB1 at a concentration of 10 ng/ml, which was comparable to the circulating HMGB1 level in active AAV patients, as demonstrated in our previous study [18], for 30 min at 37 °C. Then we stimulated the pretreated neutrophils with a concentration of patient-derived ANCA-positive IgG at 300 μg/ml in an incubator containing 5 % CO_2_ at 37 °C for 3 h. In order to investigate the role of candidate receptors through which HMGB1 exerted its effect, neutrophils were first incubated with blocking antibodies (anti-TLR2 at 10 μg/ml; anti-TLR4 at 10 μg/ml; RAGE-Fc at 1 μg/ml) for 30 min on ice. The above time set and inhibiting concentrations were according to our previous study [21]. We used phorbol myristate acetate (PMA) at a concentration of 100 nM in an incubator containing 5 % CO_2_ at 37 °C for 3.5 h as the positive control. Then we fixed the cells with 4 % paraformaldehyde (PFA) for further immunocellular chemistry (ICC).

### NETs immunofluorescence

We detected NETs by immunolabeling as described previously [26]. Coverslips with the fixed cells were removed from the plates and processed by floating on drops kept on a parafilm sheet covering a test tube stand. The samples were permeabilized for 1 min with 0.5 % Triton X-100 after washing with PBS, washed again with PBS. Unspecific binding sites were blocked in PBS containing 5 % donkey serum. The samples were then incubated for 60 min respectively with the primary antibody as follows: anti-human myeloperoxidase mouse monoclonal antibody (diluted 1:100) and anti-human Cit-H3 rabbit polyclonal antibody (diluted 1:100). After washing with PBS, each primary antibody was visualized using secondary antibodies coupled to AF488-labeled donkey anti-rabbit IgG and Cy3-labeled donkey anti-mouse IgG (both 1:400, Jackson ImmunoResearch, West Grove, PA, USA) were applied for 60 min. The primary and secondary antibodies were diluted with PBS. After incubation for 60 min with the secondary antibodies, the specimens were washed with PBS, and the DNA was stained with DAPI for 5 min. All procedures were performed at room temperature. In negative controls, primary antibodies were replaced by PBS. Confocal images were captured with a Zeiss LSM 780 confocal microscope (Zeiss, Jena, Germany). Five images taken randomly from different regions of each coverslip in the experiment were taken with the 10× lens on a fluorescence microscope [25]. Exposure times of each channel were kept constant over the whole series in the experiment after calibrating on a bright representative sample to avoid saturated pixels. The image files were analyzed with the Image-Pro Plus 6.0 software (Media Cybernetics, Silver Spring, MD, USA). The NET percentage was calculated as follows: NET rate [%] = 100 × number of neutrophils displaying expanded nuclei and releasing DNA fibers/total number of neutrophils.

### Quantification of DNA release from neutrophils

NETs formation was quantified using PicoGreen as described previously [27]. Lambda DNA of known concentration was serially diluted with Tris-EDTA (TE) buffer (10 mM Tris-HCl, 1 mM EDTA, pH 7.5) to create standard DNA samples. To follow NETs formation, 100 μl fresh neutrophils (1 × 10^5^cells) were seeded in Costar 96-well black plates (Corning, Tewksbury, MA, USA) in the presence of 0.5 % FBS. Neutrophils were pretreated with buffer or 10 ng/ml HMGB1 for 30 min followed by stimulation with MPO-ANCA-positive IgG, or PR3-ANCA-positive IgG, or normal IgGs in an incubator containing 5 % CO_2_ at 37 °C for 3 h, respectively. For assay of the role of candidate receptors, certain groups of neutrophils were preincubated with relevant reagents for 30 min on ice before being pretreated with HMGB1. Some 100 μl of the PicoGreen dye diluted 1:200 in TE buffer was added to the microplate in order to make a final volume of 200 μl per well. Following incubation in the dark for 5 min at room temperature, the fluorescent signal of the sample was measured using the microplate fluorescence reader (TriStar Multimode Microplate Reader LB941, Berthold Technologies, Bad Wildbad, Germany), at an excitation wavelength of 480 nm and an emission wavelength of 530 nm.

### Statistical analysis

Differences between the two sets of data were analyzed using *t* tests. When the differences between more than two sets of data were analyzed, we used the one-way analysis of variance. A *P* value < 0.05 was considered to be statistically significant. Reported values were expressed as mean ± standard deviation (SD). Analyses were performed on SPSS version 13.0 for Windows (SPSS Inc, Chicago, IL, USA).

## Results

### Neutrophils pretreated with HMGB1 showed greater ability to produce NETs in the presence of ANCA

**Fig. 1:**
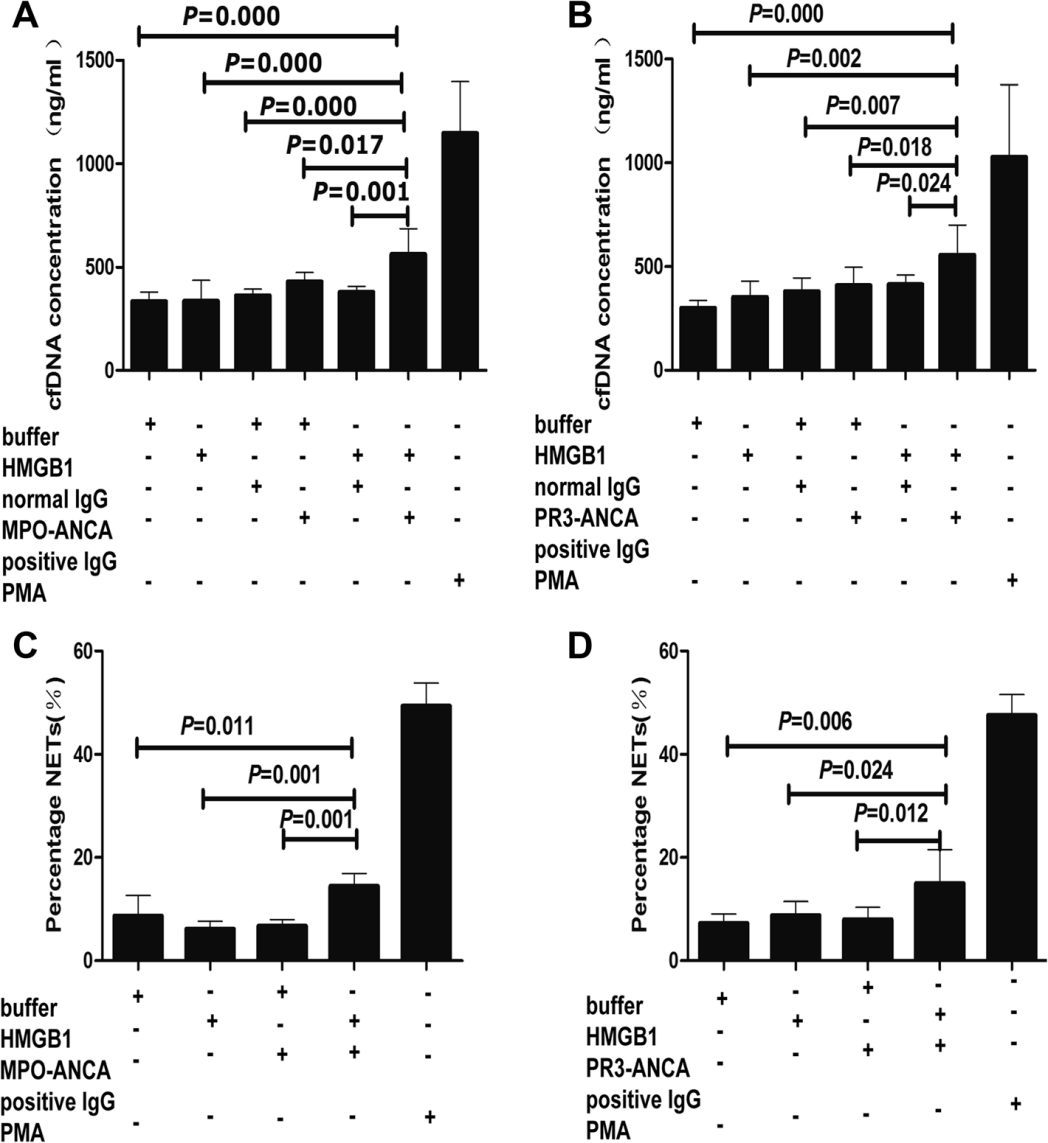
Neutrophils pretreated with HMGB1 showed greater ability to produce NETs in the presence of ANCA-positive IgG. NETs induced by MPO-ANCA-positive IgG (**a**) or PR3-ANCA-positive IgG (**b**) were measured by cell-free DNA concentration in HMGB1-pretreated neutrophils. **c** and **d** were representative histograms showing that the percentage of NETs formation induced by MPO-ANCA-positive IgG and PR3-ANCA-positive IgG in HMGB1-pretreated neutrophils. *Bars* represent mean ± SD of repeated measurements on neutrophils of 9–12 independent experiments and 9 donors. *ANCA* antineutrophil cytoplasmic antibody, *HMGB1* high-mobility group box 1, *IgG* immunoglobulin G, *MPO* myeloperoxidase, *NETs* neutrophil extracellular traps, *PR3* proteinase 3, PMA phorbol myristate acetate

We investigated the effects of HMGB1 on ANCA-induced NETs formation. ANCA-IgG were prepared from two patients with active PR3-ANCA-positive vasculitis, five patients with active MPO-ANCA-positive vasculitis and three healthy volunteers, respectively. Neutrophils of the abovementioned nine healthy donors were analyzed. The NETs were quantified by measuring cell-free DNA (cfDNA) concentration with the Quant-iT PicoGreen fluorescence probe. Compared with the buffer control, the cell-free DNA concentration increased significantly in neutrophils incubated with HMGB1 plus MPO-ANCA-positive IgG or PR3-ANCA-positive IgG (334.09 ± 46.89 vs. 563.32 ± 122.07, *P* < 0.001; 303.44 ± 37.14 vs. 563.79 ± 145.94, *P* < 0.001, respectively). Compared with neutrophils incubated with HMGB1 or ANCA-positive IgG alone, the cell-free DNA concentration of NETs increased significantly from 337.29 ± 99.06 ng/ml and 430.05 ± 43.79 ng/ml to 563.32 ± 122.07 ng/ml in the neutrophils incubated with HMGB1 plus MPO-ANCA-positive IgG (*P* < 0.001, *P* = 0.017, respectively), from 359.82 ± 76.86 ng/ml and 407.25 ± 89.90 ng/ml to 563.79 ± 145.94 ng/ml in the neutrophils incubated with HMGB1 plus PR3-ANCA-positive IgG (*P* = 0.002, *P* = 0.018, respectively) (Fig. 1). No obvious NET formation was observed with HMGB1, ANCA-IgG alone, normal IgG alone or HMGB1 plus normal IgG, either. PMA was used as a positive control.

**Fig. 2:**
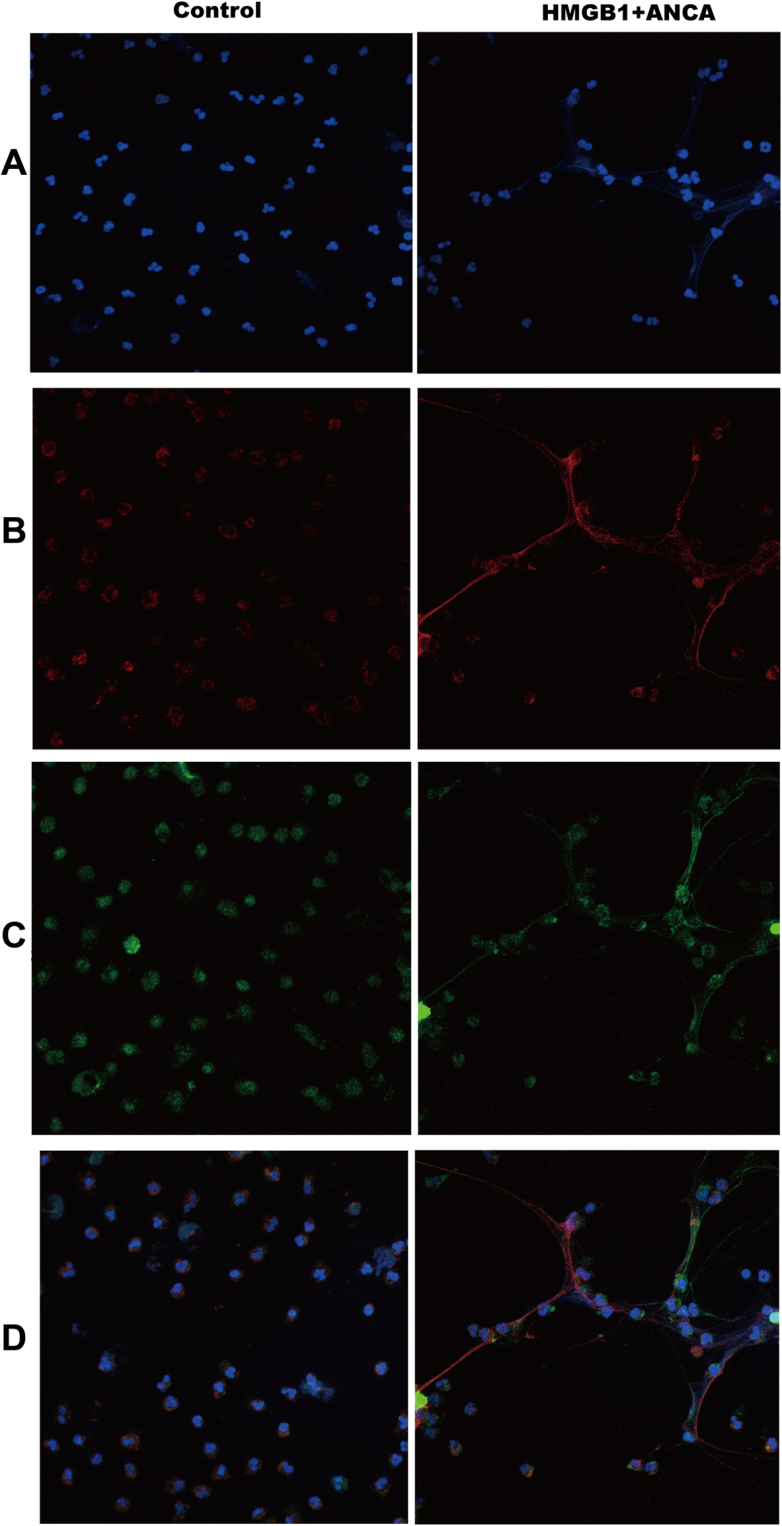
HMGB1 plus ANCA-positive-IgG induces NETs formation in vitro. Human neutrophils were activated in vitro and then processed for immunofluorescence. **a** Representative images show neutrophil DNA (*blue*). **b** Representative images show immunostained with anti-human myeloperoxidase antibodies against myeloperoxidase (*red*). **c** Representative images show immunostained with anti-Cit-histone H3 antibodies against the histone H3 complex (*green*). **d** Representative images show merged neutrophil DNA (*blue*), histone H3 (*green*) and myeloperoxidase (*red*) staining. *ANCA* antineutrophil cytoplasmic antibody, *HMGB1* high-mobility group box 1, *IgG* immunoglobulin G, *NETs* neutrophil extracellular traps

We further measured the percentage of NETs formation by immunofluorescence. The typical NETs was composed of extracellular DNA and colocalization of histone, granular proteins MPO (Fig. 2). Consistently, for MPO-ANCA-positive IgG, the percentage of NETs formation was 14.41 ± 2.48 % in the neutrophils incubated with HMGB1 plus MPO-ANCA-positive IgG, which was significantly higher than neutrophils incubated with HMGB1 alone or MPO-ANCA-positive IgG alone (6.16 ± 1.52 % vs. 14.41 ± 2.48 %, *P* = 0.001; 6.80 ± 1.21 % vs. 14.41 ± 2.48 %, *P* = 0.001, respectively). For PR3-ANCA-positive IgG, the percentage of NETs formation was 14.99 ± 6.55 % in the neutrophils incubated with HMGB1 plus PR3-ANCA-positive IgG, which was significantly higher than neutrophils incubated with HMGB1 alone or PR3-ANCA-positive IgG alone (8.82 ± 2.66 % vs. 14.99 ± 6.55 %, *P* = 0.024; 8.04 ± 2.33 % vs. 14.99 ± 6.55 %, *P* = 0.012, respectively) (Fig. 1). There was no significant difference between neutrophils incubated with ANCA-positive IgG alone and HMGB1 alone. As the positive control, 49.33 ± 4.42 % of neutrophils incubated with PMA produced NETs.

### The effects of HMGB1 and ANCA on NETs formation were dose dependent

**Fig. 3:**
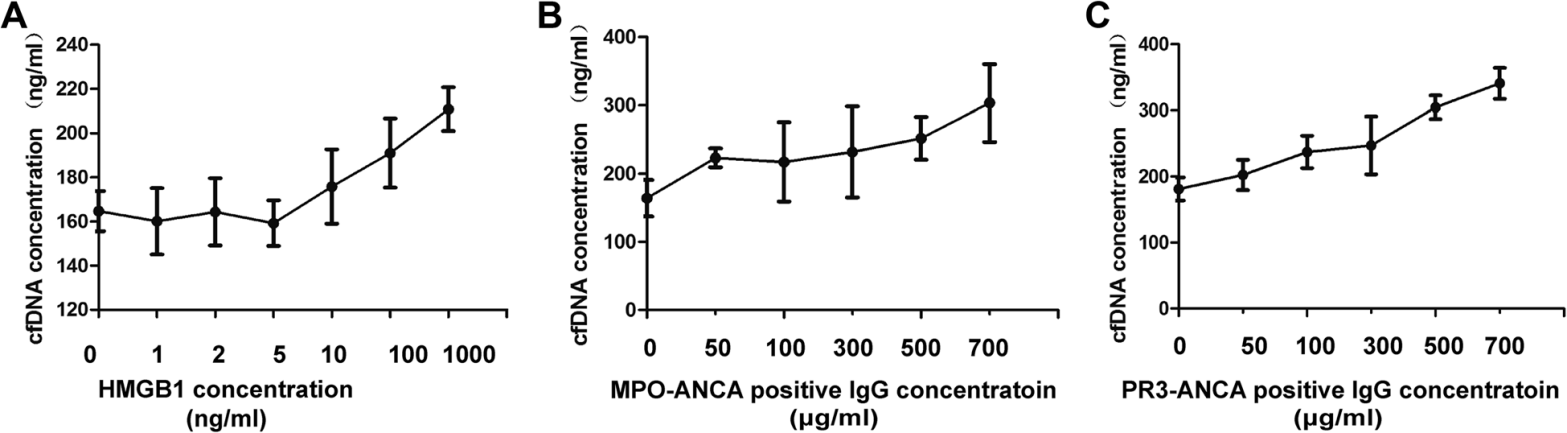
Dose–response curve of HMGB1 and ANCA-positive IgG on NETs information. **a** Dose–response curve for HMGB1 on potentiating ANCA-inducing NETs formation. **b** Dose–response curve for MPO-ANCA-positive IgG-inducing NETs formation. **c** Dose–response curve for PR3-ANCA-positive IgG-inducing NETs formation. *Bars* represent mean of repeated measurements on neutrophils of 4 independent experiments. *ANCA* antineutrophil cytoplasmic antibody, *HMGB1* high-mobility group box 1, *IgG* immunoglobulin G, *MPO* myeloperoxidase, *NETs* neutrophil extracellular traps, *PR3* proteinase 3

Neutrophils were pretreated with various concentrations of HMGB1 (0, 1, 2, 5, 10, 100 and 1000 ng/ml, respectively), then were stimulated with ANCA-positive IgG at a concentration of 300 μg/ml. The results showed that the effect of HMGB1 potentiating ANCA-inducing NETs formation was dose dependent (Fig. 3a).

On the other hand, neutrophils were pretreated with the same concentrations of HMGB1 (10 ng/ml), then were stimulated by various concentration of ANCA-positive IgG (0, 50, 100, 300, 500, and 700 μg/ml, respectively). The results showed that the effects of PR3- and MPO-ANCA-positive IgG-inducing NETs formation were both dose dependent (Fig. 3b and c).

### HMGB1-dependent engagement of TLR2, TLR4 and RAGE contributed to NETs formation in the presence of ANCA

**Fig. 4:**
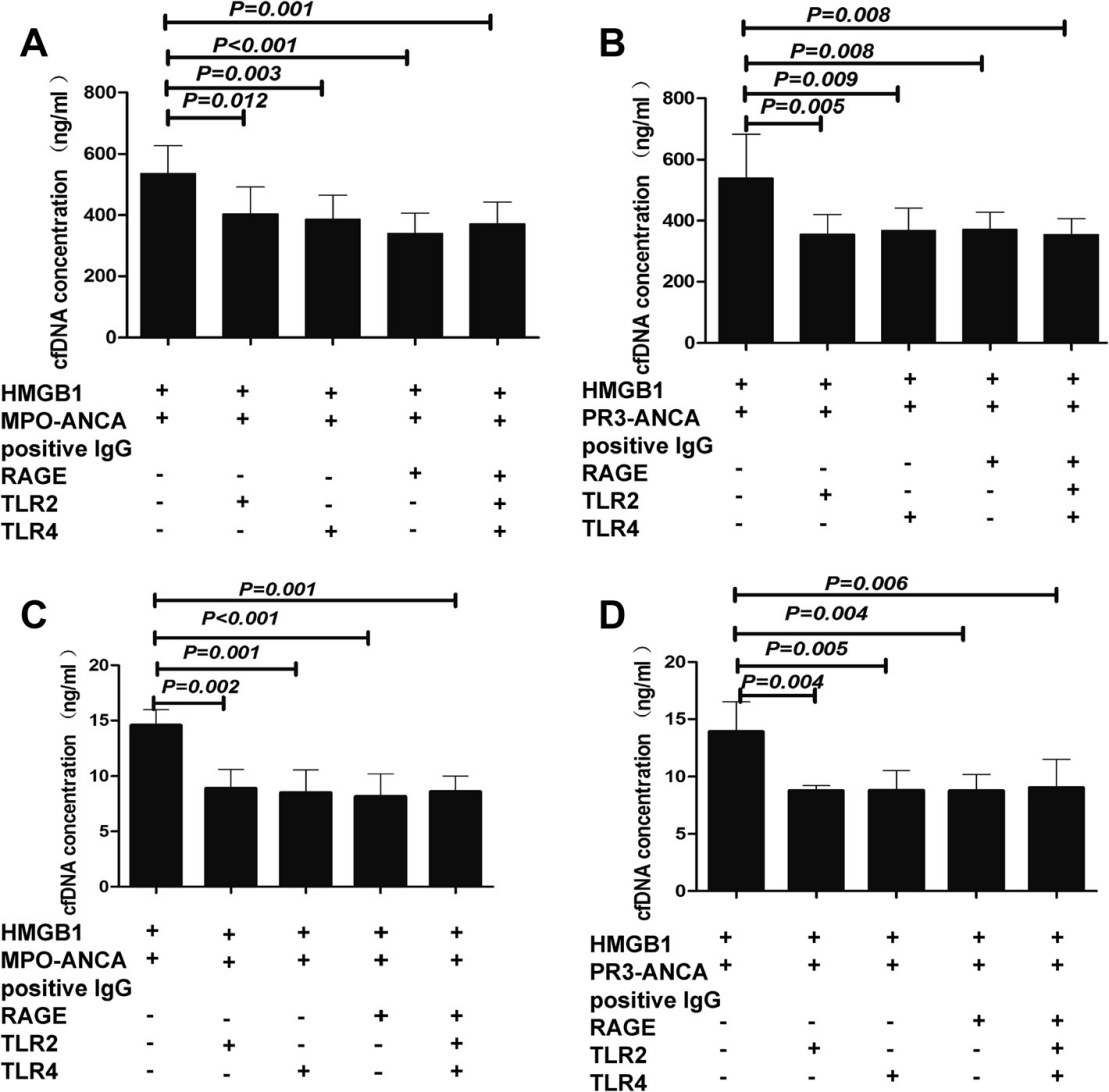
HMGB1-dependent engagement of TLR2, TLR4 and RAGE contributed to NETs formation in the presence of ANCA-positive IgG. Blockage of TLR2, TLR4 and RAGE decreased DNA release by MPO-ANCA-positive IgG (**a**) or PR3-ANCA-positive IgG (**b**) in HMGB1-pretreated cells. Blockage of TLR2, TLR4 and RAGE decreased percentage of NETs formation by MPO-ANCA-positive IgG (**c**) or PR3-ANCA-positive IgG (**d**) in HMGB1-pretreated cells. *Bars* represent mean ± SD of repeated measurements on neutrophils of 8–9 independent experiments and 9 donors. *ANCA* antineutrophil cytoplasmic antibody, *HMGB1* high-mobility group box 1, *IgG* immunoglobulin G, *MPO* myeloperoxidase, *NETs* neutrophil extracellular traps, *PR3* proteinase 3, *RAGE* receptor for advanced glycation end products, *TLR* Toll-like receptor

Since HMGB1 contributes to NETs formation in the presence of ANCA-positive IgG, we next investigated whether TLR2, TLR4 and RAGE were required in the process of HMGB1 promoting ANCA-induced NETs formation. Certain groups of neutrophils were pretreated with blocking relevant antibodies before the incubating with HMGB1. In neutrophils incubated with HMGB1 plus MPO-ANCA-positive IgG, the cell-free DNA concentration was 537.25 ± 90.11 ng/ml, which decreased to 403.51 ± 87.89 ng/ml upon preincubating with anti-TLR2 antibody (*P* = 0.012), or 386.18 ± 79.14 ng/ml upon preincubating with anti-TLR4 antibody (*P* = 0.003), or 340.62 ± 66.44 ng/ml by preincubating with RAGE antagonist (*P* < 0.001), or 371.89 ± 70.22 ng/ml by preincubating with the three blocking antibodies and inhibitors combined (*P* = 0.001). For PR3-ANCA-positive IgG, the cell-free DNA concentration was 540.33 ± 142.82 ng/ml, which was decreased to 355.79 ± 64.70 ng/ml upon preincubating with anti-TLR2 antibody (*P* = 0.005), or 367.42 ± 73.51 ng/ml upon preincubating with anti-TLR4 antibody (*P* = 0.009), or 371.46 ± 56.36 ng/ml by preincubating with RAGE antagonist (*P* = 0.008), or 355.24 ± 51.29 ng/ml by preincubating with the three blocking antibodies and inhibitors combined (*P* = 0.008) (Fig. 4).

We further measured the percentage of NETs formation by immunofluorescence. Consistently, in neutrophils incubated with HMGB1 plus MPO-ANCA-positive IgG, the percentage of NET formation decreased from 14.56 ± 1.42 % to 8.87 ± 1.75 % upon preincubating with anti-TLR2 antibody (*P* = 0.002), or 8.51 ± 2.06 % upon preincubating with anti-TLR4 antibody (*P* = 0.001), or 8.16 ± 2.03 % by preincubating with RAGE antagonist (*P* < 0.001), or 8.59 ± 1.43 % by preincubating with the three blocking antibodies and inhibitors combined (*P* = 0.001). For PR3-ANCA-positive IgG, the percentage of NETs formation decreased from 13.91 ± 2.61 % to 8.78 ± 0.44 % upon preincubating with anti-TLR2 antibody (*P* = 0.004), or 8.81 ± 1.72 % upon preincubating with anti-TLR4 antibody (*P* = 0.005), or 8.78 ± 1.42 % by preincubating with RAGE antagonist (*P* = 0.004), or 9.05 ± 2.43 % by preincubating with the three blocking antibodies and inhibitors combined (*P* = 0.006) (Fig. 4).

To further confirm the receptors through which HMGB1 exerts its effects, we used TLR2−/− and TLR4−/− mice [28]. However, RAGE−/− mice are not commercially available. These results were in line with our data of human neutrophils with inhibitors and blocking antibodies to block the activity of corresponding receptors. For detailed information, see Additional file 1.

As shown in Figure S1 in Additional file 2, compared with nonstimulated murine neutrophils, the percentage of NETs formation was significantly higher in neutrophils from B6 or B10 wild-type mice stimulated with HMGB1 plus anti-MPO IgGs (25.89 ± 1.67 % vs. 29.95 ± 2.10 %, *P* = 0.020; 13.83 ± 2.15 % vs. 19.70 ± 1.45 %, *P* < 0.001, respectively), but there were no significant differences in the percentage of NETs formation in neutrophils from TLR2−/− mice or TLR4−/− mice, between nonstimulated murine neutrophils and neutrophils stimulated by HMGB1 plus anti-MPO IgGs (26.97 ± 0.76 % vs. 26.69 ± 1.80 %, *P* = 0.930; 11.93 ± 1.48 % vs. 10.80 ± 1.91 %, *P* = 0.645, respectively).

Collectively, these results indicated that TLR2, TLR4 and RAGE were required in the process of HMGB1 promoting NETs formation in the presence of ANCA-positive IgG.

### NET formation was dependent on NADPH oxidase in the presence of HMGB1 and ANCA-positive IgG

**Fig. 5:**
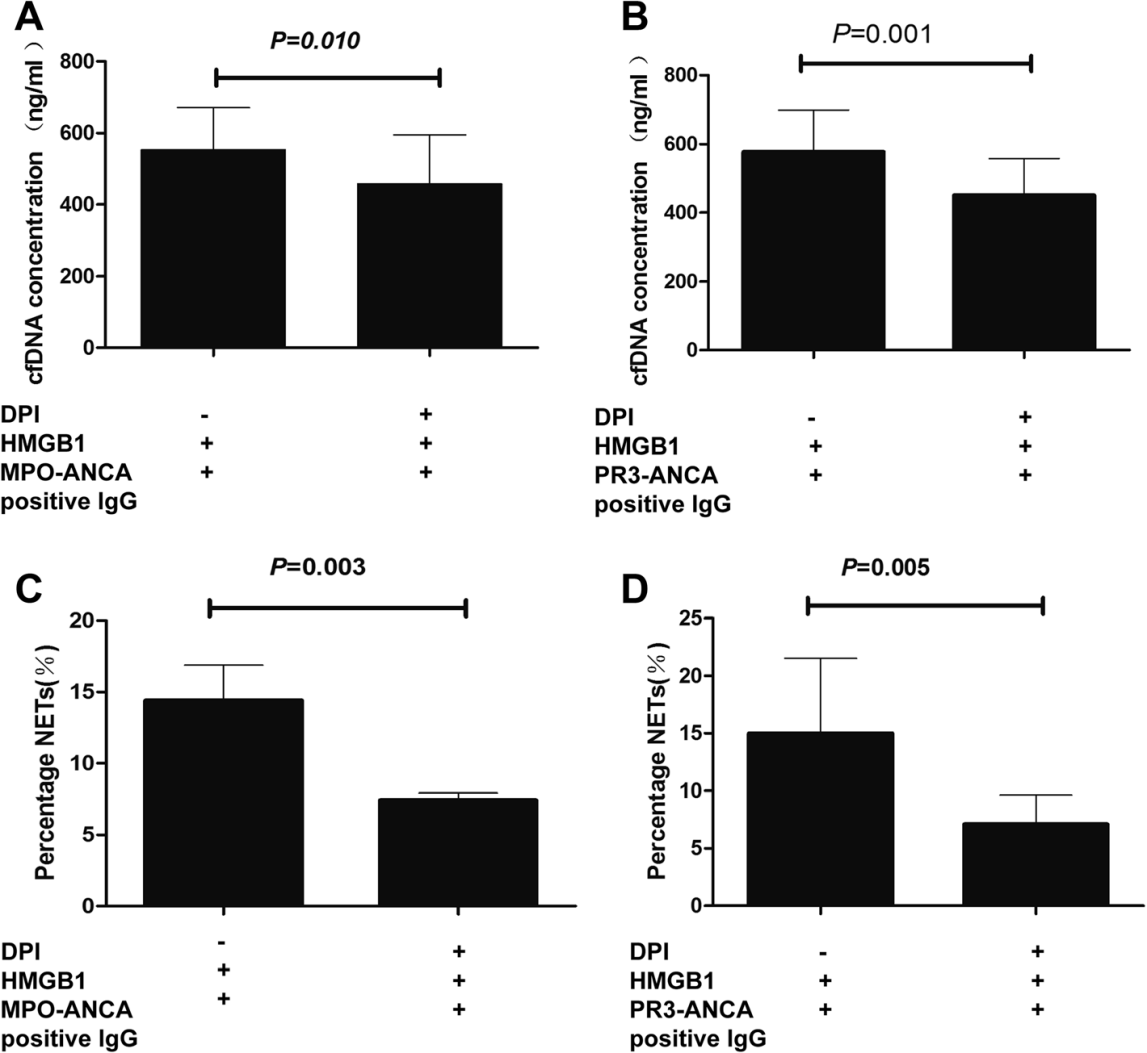
NETs formation was dependent on NADPH oxidase in the presence of HMGB1 and ANCA-positive IgG. Blockage of NADPH oxidase decreased DNA release by MPO-ANCA-positive IgG (**a**) or PR3-ANCA-positive IgG (**b**) in HMGB1-pretreated cells. Blockage of NADPH oxidase decreased percentage of NETs formation by MPO-ANCA-positive IgG (**c**) or PR3-ANCA-positive IgG (**d**) in HMGB1-pretreated cells. *Bars* represent mean ± SD of repeated measurements on neutrophils of 13–14 independent experiments and 9 donors. *ANCA* antineutrophil cytoplasmic antibody, *DPI* diphenyleneiodonium, *HMGB1* high-mobility group box 1, *IgG* immunoglobulin G, *MPO* myeloperoxidase, *NETs* neutrophil extracellular traps, *PR3* proteinase 3

Recent studies demonstrated that NETosis can occur in a reactive oxygen species (ROS)-independent manner, such as *Candida albicans* and uric acid-inducing NETs formation [29, 30]. In addition, a study by Tadie et al. showed that HMGB1 induced NETs formation also independent of NADPH oxidase ROS production [28]. Our study showed that neutrophils incubated with HMGB1 plus MPO-ANCA-positive IgG or PR3-ANCA-positive IgG, the cell-free DNA concentration decreased from 553.66 ± 118.10 ng/ml and 577.93 ± 121.69 ng/ml to 458.33 ± 136.59 ng/ml and 450.93 ± 107.54 ng/ml, respectively, by preincubating with DPI (*P* = 0.010 and *P* = 0.001, respectively) (Fig. 5). Consistently, in neutrophils incubated with HMGB1 plus MPO-ANCA-positive IgG or PR3-ANCA-positive IgG, the percentage of NET formation was decreased from 14.41 ± 2.48 % and 14.99 ± 6.55 % to 7.42 ± 0.51 % and 7.09 ± 2.57 %, respectively, by preincubating with DPI (*P* = 0.003 and *P* = 0.005, respectively) (Fig. 5). These results suggested that NETs formation in neutrophils incubated with HMGB1 plus ANCA was also dependent on NADPH oxidase ROS production.

## Discussion

In the study by Tadie et al., it was shown that HMGB1 alone contributes to NETs formation in vitro or under relevant in vivo conditions, but the concentrations of HMGB1 was 300 ng/ml in their study, which is much higher than the pathophysiological concentration of circulating HMGB1 in active AAV patients [28]. In the current study, we demonstrated that 10 ng/ml HMGB1 can contribute to ANCA-induced NETs formation. Our previous study found that HMGB1 could prime neutrophils, and further result in the respiratory burst and degranulation in the presence of ANCA [21]. The findings of the current study extended the role of HMGB1 in activating neutrophils, and thus, may contribute to the development of AAV. Although the concentration of HMGB1 used in our study was comparable to the circulating HMGB1 level in active AAV patients [18], we cannot exclude that local concentrations of HMGB1 may be higher.

Extracellular HMGB1 induces several responses, including the release of pro-inflammatory cytokines, cell proliferation and cell migration [31, 32]. Several receptors have been implicated in HMGB1-mediated functions, including RAGE and TLR2 and TLR4 [33–36]. It is not fully clear which of these receptors are required for the different bio-function of HMGB1. The current study showed that HMGB1-dependent engagement of TLR2, TLR4 and RAGE contributed to ANCA-induced NETs formation. Our previous study found that HMGB1 exerts priming effects on neutrophils by increasing ANCA antigens translocation, which was TLR4 and RAGE dependent, not TLR2 dependent [21], while Tadie’s study showed that the NETs formation induced by HMGB1 alone was TLR4 dependent, not TLR2 or RAGE dependent [28]. Thus, the process of HMGB1 potentiating ANCA-induced NETs formation may be distinct from facilitating neutrophil respiratory burst and directly inducing NETs formation.

The mechanisms responsible for NETs formation is not completely delineated yet. Despite that previous studies showed that NETs formation requires activation of NADPH oxidase and production of ROS [37], there is now growing evidence suggesting that some stimuli induce NETs formation independent of NADPH oxidase [30]. Indeed, the study by Tadie et al. showed that HMGB1-inducing NETs formation was independent of ROS generated by NADPH oxidase [28], which is consistent with rapid NETs formation in response to *Candida albicans* and uric acid-inducing NETs formation [29, 30]. However, our results indicated that the HMGB1 plus ANCA-IgG-inducing NETs formation was dependent on ROS generation. These findings suggested that HMGB1 contributing to NETs formation may involve heterogeneous mechanisms in the context of the dependency on NADPH oxidase and production of ROS.

## Conclusions

Our study demonstrated that HMGB1 can potentiate ANCA-inducing NETs formation. HMGB1 exerts effects on NETs formation through the interaction with TLR2, TLR4 and RAGE, and the process is NADPH oxidase dependent. Blockade of HMGB1 might limit inflammatory damage caused by ANCA-induced NETs formation.

## Additional files

Additional file 1:
**Materials and methods.** (PDF 194 kb) Additional file 2: Figure S1. Anti-MPO IgGs-induced NETs formation in HMGB1-pretreated murine neutrophils from TLR2–/– and TLR4–/– mice. NETs formation was measured by Sytox Green staining. The percentage of Sytox-positive cells did not increase in neutrophils from TLR2–/– mice (*A*) and TLR4–/– mice (*B*) as wild-type mice, respectively. *Bars* represent mean ± SD of repeated measurements on neutrophils of 3–6 independent experiments and mice. (TIF 11111 kb)

## Abbreviations

AAV: ANCA-associated vasculitis
ANCA: Antineutrophil cytoplasmic antibody
EGPA: Eosinophilic granulomatosis with polyangiitis
FBS: Fetal bovine serum
GPA: Granulomatosis with polyangiitis
HMGB1: High-mobility group box 1
IgG: Immunoglobulin G
IL: Interleukin
MPA: Microscopic polyangiitis
MPO: Myeloperoxidase
NETs: Neutrophil extracellular traps
NF-κB: Nuclear factor kappa B
PBS: Phosphate-buffered saline
PR3: Proteinase 3
RAGE: Receptor for advanced glycation end products
ROS: reactive oxygen species
TLR: Toll-like receptor
TNF-α: Tumor necrosis factor alpha
