# Supplementary material for: High-mobility group box 1 potentiates antineutrophil cytoplasmic antibody-inducing neutrophil extracellular traps formation
Source: Arthritis Res Ther. 2016 Jan 6;18:2. doi: 10.1186/s13075-015-0903-z (PMC4718033; doi:10.1186/s13075-015-0903-z)
Supplement: Additional file 1: — Materials and methods. (PDF 194 kb) [file 13075_2015_903_MOESM1_ESM.pdf]

Additional file

Materials and Methods

Reagents

Recombinant HMGB1 proteins were purchased from R&D Systems (Abingdon, UK). The endotoxin level of HMGB1 was below the detection limit (0.125 EU/mL) of the Limulus assay (Sigma-Aldrich, St Louis, USA). Purified anti-myeloperoxidase (MPO) rabbit polyclonal antibody (ab9535) was purchased from Abcom (Cambridge, UK), reacted with both mouse and human. Phorbol myristate acetate (PMA) was purchased from Sigma-Aldrich (St. Louis, MO). The cell-impermeable DNA dye Sytox green (S7020) was from purchased from Invitrogen (Grand Island, NY).

Mice

TLR4-deficient mice (B6.B10ScN-*Tlr4*$^{lps\text{-}del}$) mice, as well as control wild-type mice (C57BL/10) and TLR2-deficient mice (B6.129-Tlr2^{tm1Kir}) were purchased from the Model Animal Research Center of Nanjing University. Wild-type C57BL/6 was purchased from Junkeyuan Laboratory Animal Center Beijing. The mice were bred and housed in the animal facility at Peking University First Hospital. They were kept under standard conditions of temperature and light, and were fed laboratory chow and water ad libitum.

Mice neutrophil isolation

Bone marrow neutrophil isolation as described previously[1]. Mice were sacrificed, and their bone marrow from femurs and tibias was layered onto discontinuous Percoll (Sigma-Aldrich, Ontario, Canada) gradients of 80%/65%/55% and centrifuged for 30

min at 800 g at 4 ℃. Mature neutrophils, found at 80% and 65% interface, were collected. Routinely, the purity of the neutrophils was above 80%. Neutrophils were washed with PBS (Chemical reagents, Beijing, China). Neutrophils were then suspended in RPMI 1640 containing 0.5% heat-inactivated fetal bovine serum (FBS).

NETs assay

Staining of extracellular DNA with Sytox Green was used to quantify NET formation as described previously [2]. Fresh mice neutrophils (1×10^5 cells) were seeded in costar 96-well black plates (Corning, MA) in the presence of 0.5% fetal bovine serum and Sytox Green (5 μM). The cells were incubated with PMA, anti-MPO lgGs or HMGB1 for indicated time at 37 ℃, and then released DNA was quantified by reading Sytox Green fluorescence at various time points. Fluorescent signal of the sample was measured using the microplate fluorescence reader (TriStar Multimode Microplate Reader LB941, Germany), at an excitation wavelength of 480 nm and an emission wavelength of 530 nm. All experiments were performed with mice older than 4 weeks. All mice were used in accordance with the Guide for the Human Use and Care of Laboratory Animals and the approval of the Peking University First Hospital Animal Care Committee.

Statistical analysis

Differences between two sets of data were analyzed using t tests. When the differences between more than two sets of data were analyzed, we used the one-way analysis of variance. A P-value <0.05 was considered to be statistically significant. Reported values were expressed as mean ±SD. Analyses were performed on SPSS

version 13.0 for Windows (Chicago, Ill, USA).

Result

To further confirm the receptors through which HMGB1 exerts its effects, we used TLR2-/- and TLR4-/- mic**e** [3, 4]. The NETs formation was monitored using the cell-impermeable DNA dye Sytox green. As shown in additional file figure S1, compared with non-stimulated murine neutrophils, the percentage of NETs formation was significantly higher in neutrophils from B6 or B10 wild type mice stimulated with HMGB1 plus anti-MPO IgGs. (25.89± 1.67% vs. 29.95±2.10%, P=0.020; 13.83±2.15% vs. 19.70±1.45%, P<0.001, respectively). There was no significant difference between neutrophils incubated with anti-MPO IgGs alone and HMGB1 alone.

For neutrophils from TLR2-/- mice and TLR4-/- mice, there were no significant differences in the percentage of NETs formation, between non-stimulated murine neutrophils and neutrohpils stimulated by HMGB1 plus anti-MPO IgGs (26.97±0.76% vs. 26.69±1.80%, P=0.930; 11.93±1.48% vs. 10.80±1.91%, P=0.645, respectively) **(additional file figure S1)**. There was no significant difference between neutrophils incubated with anti-MPO IgGs alone and HMGB1 alone.

Collectively, the results were in line with our data of human neutrophils with inhibitors and blocking antibodies to block the activity of corresponding receptors, which indicated that TLR2 and TLR4 were required in the process of HMGB1 promoting NETs formation in the presence of anti-MPO IgGs.

Reference

1. Vong, L., P.M. Sherman, and M. Glogauer, Quantification and visualization of neutrophil extracellular traps (NETs) from murine bone marrow-derived neutrophils. Methods Mol Biol, 2013. 1031: p. 41-50.
2. Lim MB, Kuiper JW, Katchky A, Goldberg H, Glogauer M. *Rac2 is required for the formation of neutrophil extracellular traps.* Journal of Leukocyte Biology. 2011. 90: p. 771-76.
3. Wang C, Wang H, Chang DY, Hao J, Zhao MH, Chen M. High mobility group box 1 contributes to anti-neutrophil cytoplasmic antibody-induced neutrophils activation through receptor for advanced glycation end products (RAGE) and Toll-like receptor 4. Arthritis Res Ther, 2015. 17: p. 64.
4. Tadie JM, Bae HB, Jiang S, Park DW, Bell CP, Yang H, Pittet JF, Tracey K, Thannickal VJ, Abraham E, Zmijewski JW. HMGB1 promotes neutrophil extracellular trap formation through interactions with Toll-like receptor 4. Am J Physiol Lung Cell Mol Physiol, 2013. 304: p. L342-49.

Figure legends

Figure S1. Anti-MPO IgGs-induced NETs formation in HMGB1 pre-treated murine neutrophils from TLR2 -/- and TLR4 -/-mice

NETs formation was measured by Sytox Green staining. The percentage of Sytox-positive cells not increase in neutrophils from TLR2 -/- mice (A) and TLR4 -/-mice (B) as wild type mice, respectively.

Bars represent mean ± SD of repeated measurements on neutrophils of 3-6 independent experiments and mice.
