# Supplementary figures and images for: High-mobility group box 1 potentiates antineutrophil cytoplasmic antibody-inducing neutrophil extracellular traps formation
Source: Arthritis Res Ther. 2016 Jan 6;18:2. doi: 10.1186/s13075-015-0903-z (PMC4718033; doi:10.1186/s13075-015-0903-z)

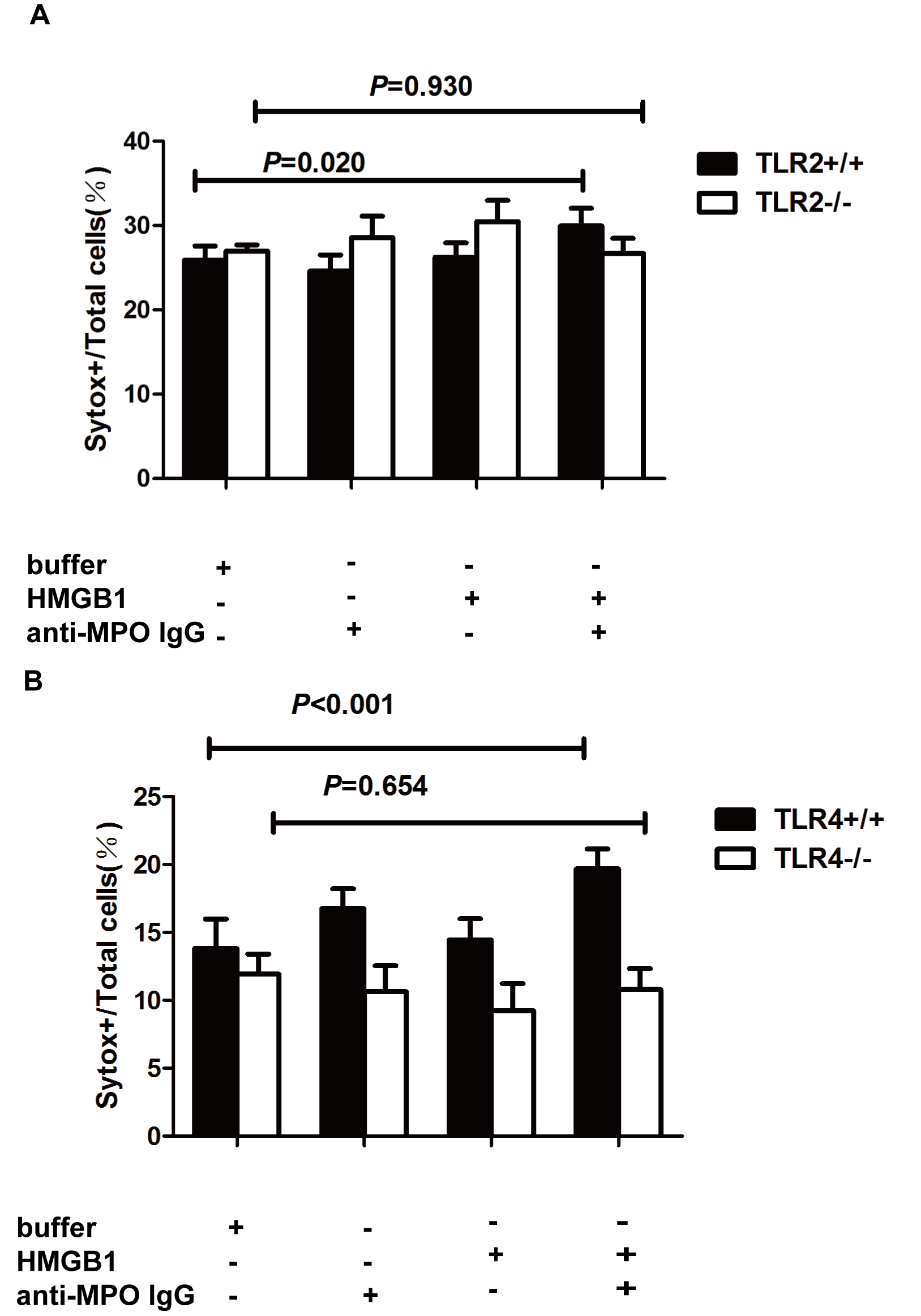

Supplement: Additional file 2: Figure S1. — Anti-MPO IgGs-induced NETs formation in HMGB1-pretreated murine neutrophils from TLR2–/– and TLR4–/– mice. NETs formation was measured by Sytox Green staining. The percentage of Sytox-positive cells did not increase in neutrophils from TLR2–/– mice (A) and TLR4–/– mice (B) as wild-type mice, respectively. Bars represent mean ± SD of repeated measurements on neutrophils of 3–6 independent experiments and mice. (TIF 11111 kb) [file 13075_2015_903_MOESM2_ESM.tif]
